# Therapeutic response and safety of the topical, sequential use of antiseptic, keratolytic, and pentamidine creams (3-PACK) on *Leishmania (Viannia) braziliensis*-infected mice

**DOI:** 10.1590/0074-02760180535

**Published:** 2019-05-13

**Authors:** Betsy Yaneth Muñoz, Julio Cesar Mantilla, Patricia Escobar

**Affiliations:** 1 Universidad Industrial de Santander, Escuela de Medicina, Centro de Investigaciones en Enfermedades Tropicales, Departamento de Ciencias Básicas, Bucaramanga, Colombia; 2 Universidad Industrial de Santander, Escuela de Medicina, Departamento de Patología, Grupo de Investigación en Patología Estructural, Funcional y Clínica, Bucaramanga, Colombia

**Keywords:** Leishmania (Viannia) braziliensis, New World cutaneous leishmaniasis, pentamidine isethionate, antiseptic, keratolytic, BALB/c mice

## Abstract

**BACKGROUND:**

Topical treatment of New World cutaneous leishmaniasis can be affected by bacterial coinfection, hyperkeratosis, and transdermal drug delivery.

**OBJECTIVE:**

The aim of this work was to evaluate the therapeutic response and safety of the topical, sequential use of antiseptic, keratolytic, and pentamidine isethionate (PMD) creams (3-PACK kit) on CL-infected BALB/c mice.

**METHODS:**

A 0.5% chlorhexidine solution (CGH), 10% salicylic acid (SA), and 3% or 6% PMD were used as antiseptic, keratolytic, and antileishmanial drugs, respectively. During the first seven days, antiseptic, followed by 10% SA gel and PMD cream, were applied topically. Subsequently, treatment was performed only with the antiseptic and PMD creams. Skin irritation, reduction of lesion size (mm^2^), and parasitic load were observed until 30 days of treatment were completed.

**FINDINGS:**

The 3-PACK treatment using 6% PMD induced a complete lesion reduction in 3/6 mice and a partial reduction in 1/6 mice, with no parasites observed. In contrast, CGH and SA alone, along with the vehicle, were not effective (p < 0.05). Moderate to severe erythema was observed at the application site.

**MAIN CONCLUSION:**

The topical 3-PACK using 6% PMD was 67% effective in the treatment of CL by *Leishmania (Viannia) braziliensis*. Currently, work is ongoing to improve PMD isethionate formulation and to determine a dose-response.

New World cutaneous leishmaniasis (NW-CL) is the most common form of leishmaniasis in Latin America.^1^ It is caused by intracellular protozoa from the genus *Leishmania* (*Viannia* and *Leishmania* subgenera) and is transmitted by the bite of *Lutzomyia* sand flies. It primarily occurs in countries such as Brazil, Colombia, Guatemala, and Peru, with roughly 67,000 new clinical cases per year.^1^ Cutaneous lesions progress from early papular and nodular lesions to an ulcerated painless nodule of various sizes, with raised edges and a granular bottom, culminating in unaesthetic scars.^2^ Cutaneous lesions are usually co-infected with bacteria, and in terms of histology, different percentages of epidermal hyperplasia and thickening of the stratum corneum (acanthosis and hyperkeratosis) have been observed.^3^ ^,^ ^4^

Treatment is recommended to prevent spread, to accelerate the healing process for slow-to-heal lesions [the spontaneous cure rate is only 6.4% for *L. (V.) braziliensis*], and to prevent disfiguring lesions or scars.^5^ ^,^ ^6^ ^,^ ^7^ ^,^ ^8^ Treatment regimens for NW-CL include pentavalent antimonials (systemic or local), oral miltefosine (MTF), pentamidine isethionate, and amphotericin B deoxycholate (or liposomal AMB).^6^ ^,^ ^8^ High cost, rate of adverse events, length of treatment, availability, and relapses are significant drawbacks for many of these treatments. Local therapies are an attractive alternative for some CL cases, especially when systemic treatment is contraindicated.^6^ ^,^ ^8^ These include thermotherapy, intralesional antimonies, and topical drug preparations such as paromomycin plus 12% methylbenzethonium or 0.5% gentamicin (called WR 279,396).^6^ ^,^ ^7^ ^,^ ^8^ ^,^ ^9^

The 3-PACK kit is a three-component treatment consisting of an antiseptic, a keratolytic, and an antileishmanial cream. Chlorhexidine digluconate (CHG), the antiseptic of choice, is a biguanide disinfectant and skin antiseptic compound that is effective against a wide range of bacteria, viruses, and yeasts.^10^ Salicylic acid (SA) is a lipid-soluble organic compound that can penetrate lipid membranes and decrease corneocyte cohesion.^11^ Pentamidine (PMD) isethionate, the selected antileishmanial drug, is a synthetic diamidine derivative and cationic drug that is commercially available as diisethionate salt (Pentacarinat^®^).^12^ PMD’s main target is thought to be the parasites’ mitochondria. Dramatic dilation, condensation, disruption of kinetoplast DNA, and collapse (decrease) of the parasites’ mitochondrial membrane potential have been demonstrated after treatment with PMD.^12^ PMD isethionate is used as a second-line therapy for treating NW-CL [except for *Leishmania (V.) guyanensis* infections, for which it is a first-line treatment],^13^ and is also recommended for the treatment of *Pneumocystis jiroveci* pneumonia and early stages of African trypanosomiasis.^14^ ^,^ ^15^ Various doses of this medication (e.g. 2-7 mg/kg/day, 1-7 doses every 2-3 days intramuscularly) have been used for NW-CL, with an overall efficacy of 35-96%.^16^ ^,^ ^17^ ^,^ ^18^ ^,^ ^19^ Systemic use of PMD isethionate has been linked to side effects such as pain, abscesses at the injection site, nausea, vomiting, malaise, myalgia, headache, hypotension, hypoglycaemia, and hyperglycaemia.^16^ ^,^ ^17^ ^,^ ^18^ ^,^ ^19^

The ultimate goal of this work is to contribute to the research on topical alternatives for NW-CL treatment; to this end, we aimed to design and evaluate the therapeutic response and safety of the consecutive topical use of an antiseptic, keratolytic, and PMD isethionate cream (3-PACK kit) on *L. (V.) braziliensis*-infected mice.

## MATERIALS AND METHODS

**Fig. 1:**
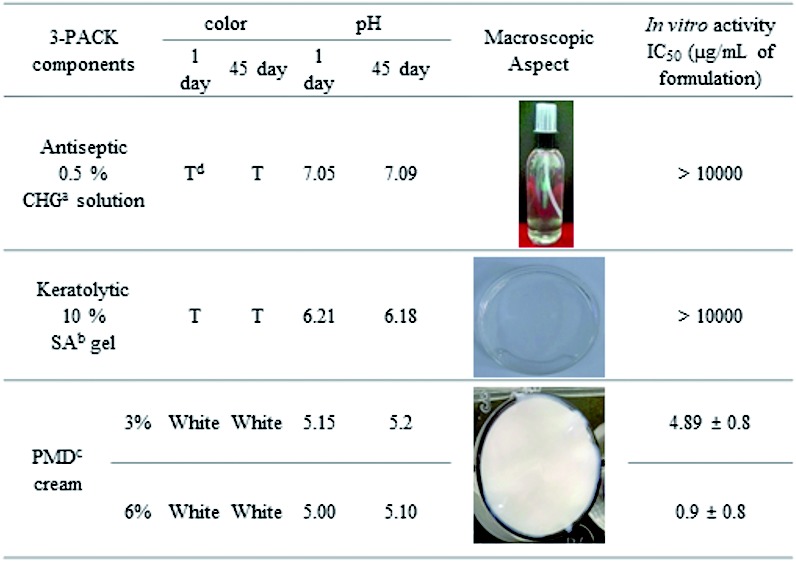
physical characteristics and *in vitro* antileishmanial activities of 3-PACK formulations, determined 1 and 45 days after preparation at ~26ºC. *In vitro* activity against *Leishmania (Viannia) braziliensis* promastigotes was determined as described in the Materials and Methods section. *a*: chlorhexidine digluconate; *b*: salicylic acid; *c*: pentamidine isethionate; *d*: translucent.

*Drugs and reagents* - Pentamidine isethionate (Pentacarinat®, PMD Sanofi-Aventis, Colombia) was supplied by the Secretary of Health of Santander, Colombia. One bottle of Pentacarinat contained 300 mg of PMD diisethionate (171 mg of PMD). Chlorhexidine digluconate (CHG), SA, and permeants (oleic acid and *trans-*β-caryophyllene) were obtained from Sigma-Aldrich (St. Louis, USA). Foetal bovine serum (FBS) and Schneider medium were purchased from Gibco® (Grand Island, NY, USA). Sodium carboxymethyl cellulose (CMC), triple stearic acid, glyceryl monostearate, triethanolamine (TEA), and glycerine were obtained from Suquín Ltda (Colombia). Physicochemical properties and chemical structures of the 3-PACK kit active agents are shown in Supplementary data (Fig. 1).

*3-PACK kit preparation* - A 0.5% (v/v) CHG solution was prepared through dilution in 70% ethyl alcohol. For the SA hydrogel, a CMC solution (2% w/v) was prepared by adding CMC powder (2.0 g) to 100 mL of distilled water and stirring at 65ºC until solubilisation was complete. After CMC dissolution, SA was added by stirring, and the solution was then homogenised for 20 min. A gel without SA was prepared as a vehicle.^20^ For the oil-in-water (o/w) PMD isethionate cream, a mixture was prepared of 4.4% triple pressed stearic acid and 1.4% Cutina MD (oily phase), and a separate mixture was prepared of TEA, glycerine, water, and excipients (aqueous phase) by melting at 70ºC. After homogenisation, the aqueous phase was added to the oil phase at the same temperature and mixed by continuous stirring for 15 min. After the emulsion was cooled to 27ºC, PMD isethionate (dissolved in DMSO) and permeants were added. In all cases, 0.5% w/v sodium benzoate was added to water as a preservative.

*Characterisation of physical parameters* - Visual appearance, colour, consistency, and homogeneity were determined. To measure pH, 2 g of each formulation was diluted in 30 mL of water, and pH was determined after 1 h using a digital pH meter (Ohaus Starter 3100, New Jersey, USA). Regarding pH, values below 7, at 7, and above 7 were considered acidic, neutral, and alkaline, respectively. The cream’s viscosity was determined using a viscometer (Brookfield DV2T, Middleboro, USA) at 26ºC, and results were expressed in centipoise (cP). Determinations were performed in triplicate. The stability of organoleptic characteristics and pH was determined at 4ºC, 26ºC, and 37ºC storage during the first 45 days after preparation.

*Parasites* - Promastigotes of *L. (V.) braziliensis* (MHOM/BR/75/M2903) were cultured at 26ºC with weekly serial passages in Schneider medium supplemented with 10% FCS.

*Promastigotes test* - Late-growth phase promastigotes at a concentration of 1x10^6^ parasites/mL were treated with serial 1:3 dilutions of PMD isethionate (in solution and cream form) for 72 h. Control parasites were maintained in medium alone or in vehicles. Inhibition of parasitic growth was determined microscopically by counting live and dead parasites with a haemocytometer. Results were expressed as the concentration needed to inhibit 50% of parasites (IC_50_), calculated by sigmoidal regression analysis using Msxlfit™ software (ID Business solution, UK).

*Mice* - BALB/c mice (8-10 weeks old) were obtained from the National Health Institute (Bogota, Colombia) and housed with a 12 h light/dark cycle at 23ºC, with water and food pellets *ad libitum*. Upon completion of the experiments, animals were anesthetised with intraperitoneal injections of 87.5 mg/kg ketamine and 12.5 mg/kg xylazine cocktail, and then euthanised by cervical dislocation.

*Ethics* - The studies were performed according to the NIH Guide for the Care and Use of Laboratory Animals. Protocols were approved by the Industrial University of Santander ethics committee (Code No. 4110).

*Skin irritation test* - This was determined in accordance with the Organization for Economic Cooperation and Development guidelines (OECD, 2015).^21^ Healthy mice (N = 2) were trichotomised and were topically treated the following day with the antiseptic, the keratolytic, and 3% or 6% PMD cream. Signs of oedema or erythema at the application site after 4, 24, and 72 h, and 9- and 14-days post-treatment were registered and scored from 0 = no irritation to 4 = severe irritation. Mice were sacrificed, and skin samples were collected for histopathological studies.

*Skin* (*keratolytic effect*) - Trichotomised healthy mice (N = 2) were treated daily for seven days with 10% SA gel or vehicle. Animals were sacrificed, and skin biopsies were collected. Changes at the application site or at the epidermal level (such as acanthosis, hyperkeratosis, and spongiosis) were determined.

*3-PACK activities in infected mice* - The following day, mice were shaved and the following day they were subcutaneously inoculated in the rump with 5×10^5^ stationary-phase promastigotes. When lesions were visible and large (15-20 weeks after infection), mice were randomly divided into four experimental groups and lesions were treated as follows: Group 1 received 3-PACK treatment using 3% PMD cream (N = 4); Group 2 received 3-PACK using 6% PMD cream (N = 6); Group 3 received 3-PACK without PMD cream (N = 2); and Group 4 received 3-PACK vehicles (N = 3). During the first seven days of treatment, antiseptic was applied each day at 5:45 pm, followed after 15 min by 10% SA keratolytic gel at 6 pm, and finally PMD cream at 6 am. Thereafter, treatment consisted only of antiseptic and PMD cream twice a day until 30 days elapsed.

Lesion size was measured weekly using a digital caliper, and area (mm^2^) was calculated. Animals were sacrificed 15 days after finishing the treatment. Impression smears from lesions, livers, and spleens were prepared on glass slides. These were air-dried, fixed with methanol, and stained with Giemsa. Biopsies were also collected for histopathological studies. Body weight and signs of skin irritation were registered each week. Per cent decrease in lesion size (%D), or improvement in each animal after treatment, was calculated according to the equation 100-[lesion size (mm^2^) after treatment x 100/lesion size (mm^2^) before treatment], and classified as significant (75-100%), moderate (50-75%), partial (25-50%), or no improvement (< 25%). In addition, parasites (amastigotes) from lesion smears and biopsies were microscopically scored as absent (−), occasionally present (+/−), or usually present (+).

*Histopathological studies* - Specimens were fixed in 10% buffered formalin (pH 7.2), embedded in paraffin, and microtomed. 5-µM sections were stained with haematoxylin and eosin, and then microscopically observed. Results were recorded with photomicrographs and the intensities of the different histopathological patterns were semi-quantitatively scored as: (-) negative, (+) discrete, (++) moderate, or (+++) intense.

*Statistical analysis* - Differences in the mean lesion sizes (mm^2^) among the groups were assessed using the Kruskal-Wallis test. P values of < 0.05 were considered statistically significant. All analyses were performed using SigmaStat software version 3.5.

## RESULTS

*Physical 3-PACK characterisation* - 3-PACK components were easily prepared and their homogeneity and stability were observed over time at different storage temperatures. The antiseptic with 0.5% CHG showed a decrease in volume at 37ºC (probably from alcohol evaporation). Cream viscosity was 147,545.50 ± 4.87 cP. Fig. 1 summarises some of the physicochemical characteristics of the 3-PACK components.

*Pentamidine parasite activity* - After 72 h of treatment, PMD isethionate (in solution) was active against *L. (V.) braziliensis* promastigotes, with IC_50_ values of 0.21 ± 0.1 µM (data not shown). Both 3% and 6% PMD creams were also active against parasites (Fig. 1), while neither 10% SA gel nor 0.5% CHG were active against parasites.

**Fig. 2:**
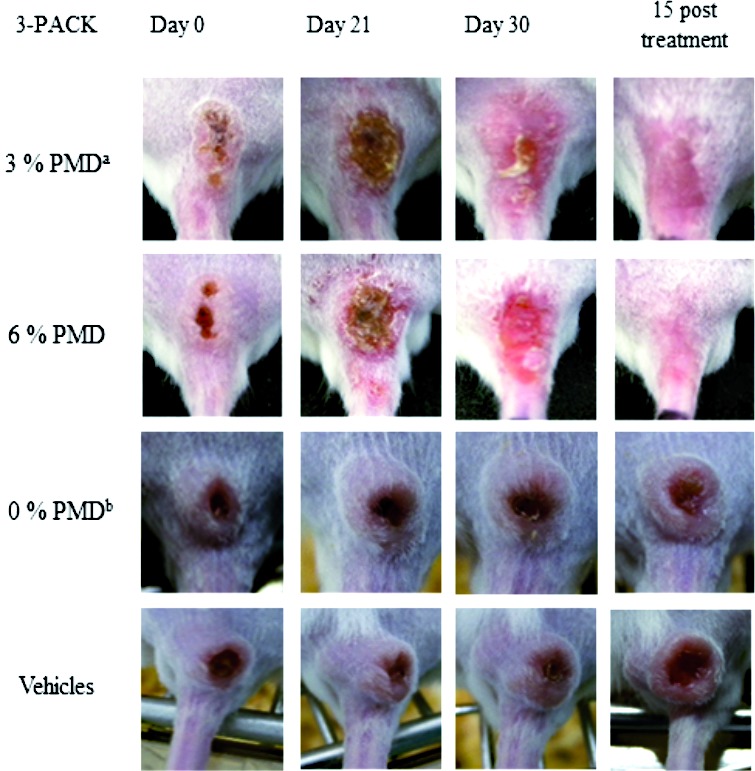
effect of 3-PACK on BALB/c mice infected with *Leishmania (Viannia) braziliensis*. Topical treatment was applied for 30 days. This image shows leishmaniasis lesions in each of the groups at the beginning, during, and 15 days after treatment. *a*: pentamidine isethionate cream; *b*: antiseptic plus keratolytic.

*Skin irritation caused by 3-PACK components in healthy skin* - No signs of oedema or erythema were observed after one dose of 0.5% CHG, 10% SA gel, and 3% or 6% PMD cream treatment at the evaluated times [Supplementary data (Fig. 2)]. Histologically, any alterations in skin biopsies were registered (data not showed).

*Keratolytic effect of 10% SA gel on healthy skin* - No signs of irritation/corrosion or redness were observed after topical application of 10% SA gel for seven days. A shedding of the stratum corneum, with no affectation on the other epidermal layers, was observed histologically after 10% SA gel treatment [Supplementary data (Fig. 3)]. In contrast, no changes in external epidermal layers were observed after treatment with vehicles [Supplementary data (Fig. 3)].

*3-PACK efficacy on CL-infected mice* - Table shows the individual percentage decreases (improvements) in lesion size and parasite loads 15 days after completing treatment. In Group 1 (3-PACK with 3% PMD), a complete healing of CL lesions with an occasional presence of parasites (+/−) was observed in one of four mice (1/4). In contrast, the 3-PACK with 6% PMD treatment (Group 2) induced a complete reduction in three of six mice and a partial reduction in one of six mice, with no parasites observed on smears and biopsies (Table). The 3-PACK treatment without PMD (CHG and SA gel only) and the vehicles were not effective, and CL lesions increased more than 20% in size, with the presence of parasites (+) in smears and biopsies of lesions (Groups 3 and 4, Table). Significant differences (p < 0.05) were found in mice treated with 3-PACK (Groups 1 and 2) compared to the controls (Groups 3 and 4), as shown in Table. Fig. 2 depicts the photographic appearance of lesions during and after 15 days of treatment. Histopathological features after 3-PACK treatment of *L. (V.) braziliensis*-infected mice are showed in Supplementary data (Fig. 4).

**TABLE:** Effect of 3-PACK treatment on cutaneous leishmaniasis (CL)-infected mice. Lesion sizes before treatment and at the end of the experiment (15 days post-treatment) were measured and the percentage decrease in lesion size (% D) was calculated. Improvement was classified as: significant (**S**, 75-100%); moderate (**M**, 50-75%); partial (**P**, 25-50%); or no improvement (**No I**, < 25%). Parasites on CL lesions after treatment were scored as absent (−), occasionally present (+/−), or usually present (+)

Group	3-PACK	Mice (M)	Lesion size (mm^2^)		Lesion (%)		Classification	Parasites
			Before	After	Increase	Decrease		
1	3% PMD^*a*^	M1	25.4	0	0	100	S	(+/−)
		M2	27.4	28.0	2.21	0	No I	(+)
		M3	13.4	46.2	32.8	0	No I	(+)
		M4	18.8	15.5	0	17.4	No I	(+/−)
2	6% PMD^*b*^	M1	33.0	0	0	100	S	(−)
		M2	64.32	46.5	0	27.66	P	(−)
		M3	171.2	0	0	100	S	(−)
		M4	186.2	0	0	100	S	(−)
		M5	22.83	70.8	48.01	0	No I	(+/−)
		M6	9.50	8.76	0	7.69	No I	(+/−)
3	0% PMD^*c*^	M1	24.19	47.5	23.31	0	No I	(+)
		M2	20.02	51.9	31.90	0	No I	(+)
4	No 3-PACK^*d*^	M1	24.91	81.4	56.52	0	No I	(+)
		M2	25.63	81.3	55.76	0	No I	(+)
		M3	32.41	85.6	53.26	0	No I	(+)

*a*: antiseptic, keratolytic and 3% pentamidine isethionate cream; *b*: antiseptic, keratolytic and 6% pentamidine isethionate cream; *c*: antiseptic plus keratolytic; *d*: vehicles. PMD: pentamidine.

*3-PACK irritation (in infected mice)* - Irritation at the treatment site (lesions) was evident on treatment day 15. A strong reddening of the lesion and signs of pain (treatment rejection and stress) were observed; grade 3 erythema corresponded to moderate to severe erythema (defined by colour and well-defined area) and grade 0 oedema corresponded to no oedema. Redness continued at a lower intensity until the end of the experiment (Fig. 2). This phenomenon was not evident when 0.5% CHG and 10% SA-gel were used. To confirm our results, a 6% PMD cream formulation was prepared under similar conditions, using PMD isethionate from Sigma-Aldrich, and tested (N = 1 infected mice). Signs of irritation were also observed 15 days after starting treatment, and consequently we decided to stop the treatment five days later (data not shown).

## DISCUSSION

In this work, a three-component (3-PACK) kit and its application protocol were designed. Each component targeted some of the most important CL lesion characteristics, such as bacterial over-infection, thickening of the epidermis (hyperkeratosis), and parasite loads inside dermal macrophages. A logical, sequential application protocol was used, starting with an antiseptic, followed by a keratolytic, and finishing with the topical application of a highly effective *in vitro* antileishmanial drug. For the antiseptic, a 0.5% CHG alcohol solution was easily prepared, stable at 4-25ºC, and non-irritating. Secondary bacterial infection is a common problem in CL and can worsen the disease by increasing infection time and exacerbating tissue destruction and scarring.^4^ Although a relationship between lesion improvement and the simultaneous use of antibiotics with antileishmanial treatment has not been demonstrated,^22^ antibiotic treatment before antimonial administration is recommended, especially for NW-CL.^4^ Additional studies are recommended to investigate CHG use for chronic or persistent wounds (as in chronic NW-CL) and its convenience compared to other treatments, i.e. neutral soap and tap water, alcohols, and antibiotics.

The use of a keratolytic agent such as SA for this NW-CL experimental model was a strategy to combat some of the epidermal histological changes present with the disease (i.e. acanthosis and hyperkeratosis) in order to increase PMD skin penetration. SA dissolved well in the CMC-hydrogel and was physicochemically stable. Topical applications of SA have been used to remove excessively cornified skin, including corns and calluses, and to treat systemic forms of hyperkeratosis, such as psoriasis and ichthyosis.^11^ In this study, 7 days of 10% SA treatment induced a reposition of the stratum corneum with no signs of skin irritation. SA was used independently of the antileishmanial drug in order to facilitate their use protocols. The balance between drug permeation and nontoxic, non-irritating, and non-allergenic effects cannot always be achieved; for example, topical paromomycin in combination with 12% methylbenzethonium chloride (a cationic quaternary ammonium permeant) are related to inflammation and pain.^23^

The third 3-PACK component was 3% or 6% PMD o/w cream formulations. PMD isethionate has been used as a drug option for NW-CL,^16^ ^,^ ^17^ ^,^ ^18^ ^,^ ^19^ and parasites from different *Leishmania* species are also susceptible (IC_50_ around 0.5-7 µM).^13^ ^,^ ^19^ The parasites used in this study were susceptible to PMD isethionate in solution at similar IC_50_ doses. For the design of the topical formulation, we used Pentacarinat (300 mg powder), which is recommended for intramuscular or intravenous application in leishmaniasis or for inhalation in the prophylaxis of *P. jiroveci* pneumonia.^14^ There is no commercially available topical PMD formulation, and it is difficult to extrapolate *in vitro* results for an *in vivo* application. We decided on an initial PMD cream concentration of 3% PMD isethionate (equivalent to 30 mg/mL of PMD isethionate) to see how it performed. Drug concentrations contained in other topical formulations tested for CL are diverse, i.e. 15% (paromomycin, WR 279,396), 5% (Imiquimod, Aldara), and 3% (amphotericin B, Anfoleish).^9^ ^,^ ^24^ ^,^ ^25^

PMD isethionate formulations in this study were easily prepared using triple pressed stearic acid and glyceryl stearate as anionic self-emulsifying bases (amply used in cosmetic and pharmaceutical skin care), and DMSO, oleic acid, and terpenes as skin penetration enhancers.^26^ A slightly acidic pH (a final pH of 5.15) was selected to prevent PMD precipitation. The PMD cream was stable at the different storage conditions used and no changes in colour and pH, or signs of phase separation, were registered. In addition, its *in vitro* antileishmanial activity was retained when it was formulated in a cream.

Once the components of the 3-PACK were prepared and characterised, we selected the scheme of use. We decided to use the antiseptic every day and the keratolytic for only seven days (to avoid the potential risk of systemic toxicity).^11^ Dosage and frequency of PMD isethionate administration were a main concern. We could not find available data on the skin permeability of PMD isethionate alone, or in conjunction with the current permeants or SA as a keratolytic. In addition, considering its physicochemical properties Supplementary data (Fig. 1), PMD did not meet all the requirements for a drug to penetrate the SC: poor absorption or permeation is more likely when the molecular weight is greater than 500 g/mol, the calculated Log P is greater than 5, and there are more than 5 H-bond donors/10 H-bond acceptors.^27^ Information about the topical use of pentamidine was scarce. A topical application of 10% PMD isethionate and 10% usnic acid cream over eight consecutive days reduced lesion size (reduction of 27.37%) and parasite numbers in *L. (L.) amazonensis*-infected hamsters, compared to controls.^28^ In addition, intralesional PMD isethionate (three injections of 120 μg/mm^2^ to the lesion area over five days) has resulted in a cure rate of 72% for Bolivian *L. (V.) braziliensis-*infected patients.^29^

In this study, we used the 3-PACK to treat well-formed CL lesions. The 3-PACK serial treatment using 3% PMD cream was able to reduce the lesion size by 100% in one out of four mice; however, 6% PMD cream was able to completely reduce CL lesions in 3/6 mice and partially reduce lesions in 1/6 mice, with no microscopically-observed parasites on stained smears and biopsies from lesions (efficacy of 67%). This is a very encouraging result, especially given the mouse model used. As seen in Fig. 2, very well defined lesions (nodule and open ulcer), similar to certain localised-CL lesions in humans, can be induced in mice after *L. (V.) braziliensis* infections. Using the same animal model, we demonstrated both treatment efficacies: 1. any cure (or *in vivo* antileishmanial effect) after topical usage of ketoconazole or liposomal chlorine aluminum phthalocyanine systems (as photosensitizer for photodynamic therapy) or 2. a complete reduction of lesion and parasite loads (by microscopic analysis) after miltefosine gel treatment on *L. (V.) braziliensis*-infected mice.^30^ ^,^ ^31^

The skin irritation observed at the application site, mainly due to the PMD isethionate cream, is a major concern. The design of a suitable PMD (isethionate or its free base form) delivery system to allow for controlled release, better absorption, higher therapeutic efficacy, and less toxicity is necessary. Different systems for PMD delivery ― such as chitosan-coated niosomes (inPentasomes), mesoporous silica nanoparticles, and sugar-grafted liposomes ― have been designed to treat leishmaniasis and other diseases, with interesting results.^32^ ^,^ ^33^

In this study, we demonstrated that 3-PACK treatment using 6% PMD isethionate cream was 67% effective in the treatment of CL caused by *L. (V.) braziliensis*. Ongoing work will improve the PMD isethionate formulation so that it is less irritating and more effective, and will also help to determine a dose-response using a larger number of animals.
