# Supplementary material for: Therapeutic response and safety of the topical, sequential use of antiseptic, keratolytic, and pentamidine creams (3-PACK) on Leishmania (Viannia) braziliensis-infected mice
Source: Mem Inst Oswaldo Cruz. 2019 May 13;114:e180535. doi: 10.1590/0074-02760180535 (PMC6516740; doi:10.1590/0074-02760180535)
Supplement: Supplementary file 1 [file 1678-8060-mioc-114-e180535-s.pdf]

Component	Physicochemical properties	Chemical structure
CHG[a]	MW: 505.447 g/mol<br>Log *P*: 4.51<br>Pka:10.52<br>Water solubility:<br>800 mg/L	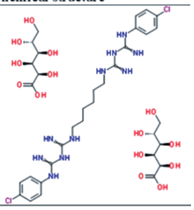
SA[b]	MW: 138.1207 g/mol<br>Log *P*: 1.98<br>Pka: 2.79<br>Water solubility: 2,240 mg/L	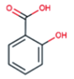
PMD isethionate[c]	MW: 592.68 g/mol<br>Log *P*: 2.32<br>Pka: 12.13<br>Water Solubility: greater than or equal to 100 mg/mL	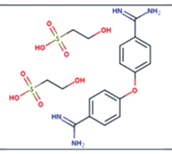

Fig. 1: physicochemical properties of 3-PACK kit active agents. *a*: chlorhexidine digluconate; *b*: salicylic acid: *c*: pentamidine isethionate.

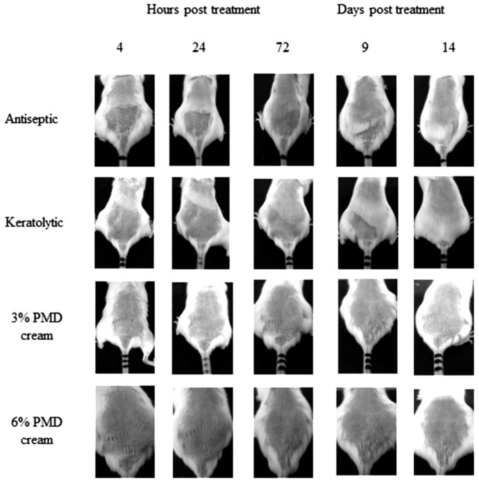

Fig. 2: skin irritation from 3-PACK components in healthy BALB/c mice. This figure shows the macroscopic characteristics of the lesions after applying a single dose of antiseptic (0.5% chlorhexidine digluconate), keratolytic (10% salicylic acid gel), or pentamidine (PMD) isethionate cream (3% and 6% PMD).

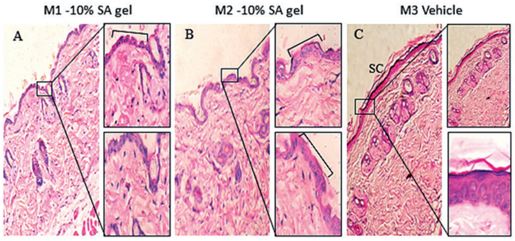

Fig. 3: keratolytic effect of 10% salicylic acid (SA) gel on skin. BALB/c mice were treated with 10% SA-gel [M1 (A), M2 (B)] or vehicle (C) for seven days. Microphotographs at different magnifications show the effect of SA on the stratum corneum (SC). A decrease or complete elimination of the SC (M1 and M2) and well-defined SC (vehicle) can be seen (square amplification).

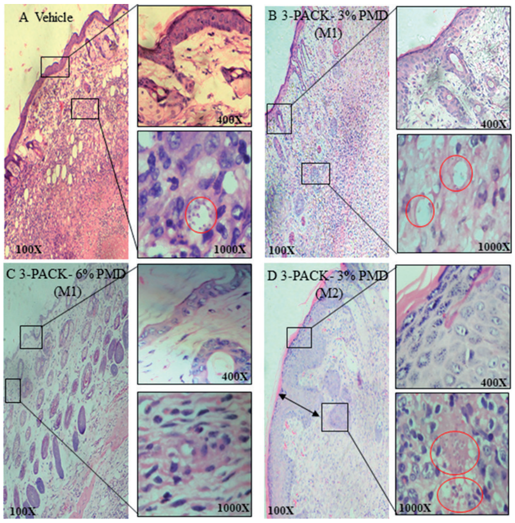

Fig. 4: histopathological features after 3-PACK treatment of *Leishmania (Viannia) braziliensis*-infected mice. The microphotographs taken at different magnifications show epidermal/dermal changes: (A) parakeratosis and abundant inflammatory infiltrate in vehicle-treated mice; (B) normal skin structure with only three amastigotes in M1 mice treated with 3-PACK-3% pentamidine (PMD), along with elimination of cutaneous leishmaniasis (CL) lesions; (C) normal skin structure and no parasites in M1 mice treated with 3-PACK-6% PMD, with elimination of CL lesions; (D) spongiosis, parakeratosis, hyperkeratosis, and acanthosis (arrow) in M2 mice treated with 3-PACK-3% PMD, with no lesion reduction. Intra- and extracellular amastigotes are indicated with red circles.

cc BY
